# 

**Published:** 1901-01-12

**Authors:** 


### NOTICE TO CORRESPONDENTS.

All MSS., Letters, Books for Review, and other matters intended for the Editor should be addressed THE EDITOR, "THE HOSPITAL," THE HOSPITAL BUILDING, 28 & 29 SOUTHAMPTON STREET, STRAND, W.C.

The Editor cannot undertake to return rejected MSS., even when accompanied by stamped directed envelope.

**Notice to Advertisers and Subscribers.**

All Advertisements, Orders for Copies of THE HOSPITAL, and Business Communications should be addressed to **THE MANAGER (Not to the Editor),** THE HOSPITAL, 28 & 29 Southampton Street Strand, London, W.C.

The Cost of Subscription, post free, is as follows:—Per week, 2½d.; per quarter, 2s. 8d.; per half-year, 5s. 3d.; per annum, 10s. 6d. Foreign:—Per annum, 15s. 2d., payable, in all cases, in advance.

**NOTICE.—Vol. XXVIII. of "The Hospital" (April to September, 1900), in handsome cloth binding, is now on sale at the Office of this Journal, price, 7s. 6d. Cases for binding the Numbers contained in this Volume 1s. 6d. Index to Vol. XXVIII., 2½d., post free.**

**The Monthly Part for January is now ready. Price 6d., by post 8d.**

### BOOKS RECEIVED.

THE CANCER SOCIETY.

Elementary Notes on Cancer." By Herbert Snow, M.D.Lond.

BAILLIÈRE, TINDALL AND COX.

"How to Avoid Tubercle." By Tucker Wise, M.D.

"The History of Ancient Gynæcology." By W. J. Stewart McKay, M.B., M.Ch., B.Sc.

"Uterine Tumours: The Pathology and Treatment." By W. Roger William, F.R.C.S.

"Syphilis of Children in Every-Day Practice." By George Carpenter, M.D.Lond.

THE HAZEL PURE FOOD COMPANY.

"How to Gain Health and Long Life."

CHATTO AND WINDUS.

"Herbert Fry's Royal Guide to London Charities." Edited by John Lane.

ADAM AND CHARLES BLACK.

"Who's Who? 1901."

"The Englishwoman's Year-Book and Directory, 1901." Edited by Emily Janes.

JOHN BALE, SONS, AND DANIELSSON.

"The British Sanatoria Annual."

SAMPSON LOW, MARSTON AND CO.

"Mosquitos and Malaria." By Cuthbert Christie, M.B. and C.M.Edin., with six full-page plates.

### SCRAPS AND GLEANINGS.

THE additions which are being made to the Coldash Cottage Hospital at a cost of £1,000 comprise new kitchens, dining-room for the matron and nurses, verandahs and offices.

OWING to the death of Prince Christian Victor, Princess Christian has cancelled her engagement to open the new workhouse hospital at Salterhebble, Halifax, in March next.

SIR W. T. LEWIS has given 10,000 shillings (£500) to the *Western Mail* Shilling Fund for the Cardiff Infirmary. The *matinée* at the Empire on behalf of the Mayor's Fund for the Cardiff Infirmary produced £431.

THE Archbishop of Canterbury has promised to preside at the annual meeting of the After-Care Association for Poor Persons Disharged Recovered from Asylums for the Insane to be held in the Library at Lambeth Palace on January 29th, at 2.30 p.m.

THE amount collected by the Manchester Hospital Saturday and Sunday Fund during the year ending June 30th last was £7,233, viz., £3,938 from the Sunday collections, and £3,294 from the Saturday collections. These amounts show a total nett decrease of £358 on last year's collections.

THE Warwick Board of Guardians has decided to erect an entirely new workhouse infirmary at a cost of £18,000. This scheme will provide accommodation for 132 patients, and its cost will not exceed £140 per bed. The Local Government Board are to be applied to for sanction to borrow the money.

THE application of the Cork Fever Hospital to the Cork Corporation for a grant of £500 to tide them over their present financial difficulties has been referred to the Public Health Committee. The Trustees of the Fever Hospital are very heavily in debt, and it is stated that their credit at the bank has been stopped.

AT the meeting of the subscribers to the Syracusa Convalescent Home for invalided soldiers it was decided to write to the P. M. O. of the army to find out how long it was likely patients would continue to be sent to the home, as it was the intention of the committee to keep the Home open only so long as soldiers were sent to Torquay. The Hon. Treasurer reported that the amount received to date had been £2,831, of which sum £1,209 had been expended, leaving, with £88 in hand, a balance of £1,642 in hand. Since the Home was opened 127 soldiers had been received.

At a recent meeting of the governors of the Cardiff Infirmary the new rules reconstituting the management were agreed to unanimously. Under these new rules working-men subscribers will no longer be excluded from the administration, which will be carried on by twenty-five members elected by the governors, two special workmen's representatives, five members of the honorary medical staff, and two lady visitors. The working men will further have a considerable voice at the annual meetings of governors, as the new rules provide that collective subscriptions will enable the subscribers to nominate one of their own number to represent them as governor.

### The Student of Medicine.

### APPOINTMENTS.

**E. G. Adams,** M.B.Lond., F.R.C.S.Eng., appointed Medical Officer for the No. 1 B District of the Langport Union. **Charles Aymer,** M.B.Aberd., C.M., appointed Medical Officer and Vaccinator to the Parish Councils of Berrie, Arbuthnott, Kinneff, and Catterline. **Reginald Threlfall Bailey,** M.R.C.S.Eng., L.R.C.P.Lond., appointed Resident Assistant Medical Officer to the Mill Road Infirmary, Liverpool. **R. W. Barnes,** M.R.C.P.I., L.R.C.S.Edin., appointed District Medical Officer of the Bridgwater Union. **George Rogden,** M.B., Ch.B.Edin., appointed House-Physician to the Salisbury Infirmary. **T. A. B. Cooke,** M.R.C.S., L.R.C.P.Lond., appointed Medical Officer of Health to the Neyland Urban District. **W. Duncan,** L.R.C.P., L.R.C.S.Edin., appointed Medical Officer for the Eythorne District of the Eastry Union, District Medical Officer of the Dover Union. **R. S. Elvins,** L.R.C.P., L.R.C.S.Edin., appointed Medical Officer to the Tollerton District of Easingwold Union. **T. Fred. Gardner,** M.R.C.P.Lond., appointed Physician to the Royal Boscombe and West Hants Hospital. **J. Hoyle,** M.R.C.S., L.R.C.P.Lond., appointed District Medical Officer of the Burnley Union. **Arthur Lloyd Jones,** M.R.C.S.Eng., D.P.H.Camb., appointed Medical Officer of Health to the Oystermouth District Council. **Oscar Lemin,** L.S.A.Lond., appointed Resident Medical Officer to the St. Marylebone General Dispensary, Welbeck Street, W. **H. B. Mapleton,** M.D.Edin., D.P.H., R.C.P.S.Lond., appointed Medical Officer of Health for the Dawlish Urban District. **James Mudge,** L.R.C.P.Edin., M.R.C.S.Eng., appointed Medical Officer of Health for the Ludgvan Urban District. **J. R. Pooler,** appointed House Surgeon to the Birmingham and Midland Ear and Throat Hospital. **Hugh Mallinson Rigby,** M.B., B.S.Lond., F.R.C.S.Eng., appointed Assistant Demonstrator of Anatomy at the London Hospital Medical College. **J. E. P. Shera,** L.R.C.P.I., L.R.C.S.I., appointed Second Assistant Medical Officer to the Kent County Asylum, Chartham, Canterbury **M. Stewart,** L.R.C.P., L.R.C.S.Edin,, appointed Certifying Factory Surgeon for the Caldbeck District of Cumberland. **Daniel L. Thomas,** M.R.C.S., L.R.C.P., D.P.H., appointed Medical Officer of Health to the Stepney Borough Council. **M. S. Walsh,** L.R.C.P., L.R.C.S.Irel., appointed Assistant Resident Medical Officer to the North Dublin Union. **W. P. Warburton,** L.R.C.P., L.R.C.S.Edin., L.F.P.S.Glasg., appointed Medical Officer of Health for the Skirlaugh Rural District. **W. B. Warrington,** M.D.Lond., M.R.C.P., appointed Assistant Physician to the Stanley Hospital, Liverpool.

